# Multiple Autoantibodies Display Association with Lymphopenia, Proteinuria, and Cellular Casts in a Large, Ethnically Diverse SLE Patient Cohort

**DOI:** 10.1155/2012/819634

**Published:** 2012-09-04

**Authors:** Rufei Lu, Julie M. Robertson, Benjamin F. Bruner, Joel M. Guthridge, Barbara R. Neas, Swapan K. Nath, Jennifer A. Kelly, Kathy L. Moser Sivils, Eliza F. Chakravarty, Diane L. Kamen, Gary S. Gilkeson, Daniel J. Wallace, Michael H. Weisman, R. Hal Scofield, John B. Harley, Judith A. James

**Affiliations:** ^1^Arthritis and Clinical Immunology, Oklahoma Medical Research Foundation, Oklahoma City, OK 73104, USA; ^2^Department of Medicine and Department of Pathology, University of Oklahoma Health Sciences Center, Oklahoma City, OK 73104, USA; ^3^Department of Biology, Harding University, Searcy, AR 72143, USA; ^4^Department of Biostatistics, University of Oklahoma Health Sciences Center, Oklahoma City, OK 73104, USA; ^5^Division of Rheumatology and Immunology, Medical University of South Carolina, Charleston, SC 29425, USA; ^6^Divison of Rheumatology, Cedars-Sinai Medical Center, Los Angeles, CA 90048, USA; ^7^US Department of Veterans Affairs Medical Center, Oklahoma City, OK 73105, USA; ^8^US Department of Veterans Affairs Medical Center, Cincinnati, OH 45220, USA; ^9^Division of Rheumatology, Cincinnati Children's Hospital Medical Center, Cincinnati, OH 45229, USA

## Abstract

*Purpose*. This study evaluates high-throughput autoantibody screening and determines associated systemic lupus erythematosus (SLE) clinical features in a large lupus cohort. *Methods*. Clinical and demographic information, along with serum samples, were obtained from each SLE study participant after appropriate informed consent. Serum samples were screened for 10 distinct SLE autoantibody specificities and examined for association with SLE ACR criteria and subcriteria using conditional logistic regression analysis. *Results*. In European-American SLE patients, autoantibodies against 52 kD Ro and RNP 68 are independently enriched in patients with lymphopenia, anti-La, and anti-ribosomal P are increased in patients with malar rash, and anti-dsDNA and anti-Sm are enriched in patients with proteinuria. In African-American SLE patients, cellular casts associate with autoantibodies against dsDNA, Sm, and Sm/nRNP. *Conclusion*. Using a high-throughput, bead-based method of autoantibody detection, anti-dsDNA is significantly enriched in patienets with SLE ACR renal criteria as has been previously described. However, lymphopenia is associated with several distinct autoantibody specificities. These findings offer meaningful information to allow clinicians and clinical investigators to understand which autoantibodies correlate with select SLE clinical manifestations across common racial groups using this novel methodology which is expanding in clinical use.

## 1. Introduction

Systemic lupus erythematosus (SLE) is a complex autoimmune disease that is characterized by diverse clinical symptoms and autoantibody production against a variety of nuclear and cytoplasmic antigens [1–3]. The occurrence and prevalence of these autoantibody specificities have been used to characterize the diverse clinical presentations of SLE. The standard screening assay for the detection of autoantibodies, and more specifically anti-nuclear antibodies (ANAs), is an indirect immunofluorescence. However, to identify more specific subsets of autoantibodies, immunodiffusion and enzyme-linked immunosorbent assays (ELISAs) are often employed. Newer screening technologies, like the Luminex bead-based assay performed with the Bio-Rad BioPlex 2200, are being introduced, which focus on performing a sensitive multiplex analysis of select autoantibody specificities allowing for high-throughput analysis with minimal human time investments or complicated human analysis and interpretation.

Previous work has shown, in many cases, that autoantibodies are present years before SLE diagnosis [4–6] and that autoantibodies usually preceded the onset of clinical symptoms [5, 7–9]. Select lupus autoantibodies are correlated with the occurrence of specific clinical symptoms. Lymphopenia is associated with anti-Ro and anti-dsDNA antibodies; while anti-chromatin antibodies are more commonly found with leukopenia [10–13]. ACR SLE renal classification criteria are strongly correlated with anti-dsDNA and anti-chromatin antibodies. Anti-ribosomal P antibodies associate with increased risk of nephritis in anti-dsDNA positive patients in a juvenile-onset SLE cohort [8, 10, 12, 14, 15]. Interestingly, anti-La antibodies are inversely related with renal and CNS involvement in SLE [11, 16]. Strong associations between anti-Ro and anti-La antibodies in SLE patients with skin manifestations have also been observed [12, 17]. Anti-RNP associates with Raynaud's phenomenon [18]. Interestingly, autoantibodies against proliferating cell nuclear antigen, while present in many systemic autoimmune diseases, are found in high titers in some SLE patients [3]. However, little is known about the clinical significance of those autoantibodies.

Select autoantibodies are also predictive of severe clinical manifestations of ACR criteria. In Canadian First Nations, anti-Sm antibodies are correlated with higher mortality [12]; while the presence of anti-Ro, anti-Sm, and anti-RNP antibodies is associated with increased disease severity in African-American female SLE patients [19]. Antibodies against dsDNA may increase prior to clinical disease flare in SLE patients [20]; while the presence of anticardiolipin antibodies is correlated with a more varied and severe clinical disease course in SLE patients [21], as well as with thrombotic events. These previous studies indicate the importance of examining the specific autoantibody profile in each SLE patient. However, these previous studies relied on precipitating levels of antibodies or historical chart data.

The goal of this study was to examine the detection of autoantibodies in a large ethnically diverse SLE patient cohort using a high-throughput multiplex bead-based assay. Secondarily, we explored whether associations existed between autoantibodies present in patient sera and the occurrence of specific SLE diagnostic criteria. 

## 2. Materials and Methods

### 2.1. Cohort Selection

A collection of 1,803 SLE patient serum samples were obtained from the Lupus Family Registry and Repository (LFRR) and the Lupus Genetics cohorts at the Oklahoma Medical Research Foundation (OMRF) on the basis of availability of serum, clinical information, and presence of 4 of 11 ACR classification criteria [22, 23] for each patient. Our study was comprised of 836 European-Americans (EA), 618 African Americans (AA), 255 Hispanics (HI), and 93 other races/ethnicities (mixed race/ethnicity, Asian, American Indian, and unknown). Within the AA SLE patients, 127 were of Gullah descent from off the coastal islands of South Carolina and Georgia. Each SLE patient previously had questionnaires, personal interviews, and standardized medical record reviews for documentation of SLE classification criteria and subcriteria [24]. Clinical, demographic, autoantibody, and therapeutic information about each patient was extracted. All participants provided informed consent and the study was approved by the OMRF Institutional Review Board.

### 2.2. Serologic Autoantibody Testing

ANA antibodies were measured using indirect immunofluorescence with *HEp-2 cells* as the substrate (IIF, INOVA Diagnostics, San Diego, CA) [5, 6, 25]. Detection of ANA at a dilution of 1 : 120 or greater was considered a positive result. The ANA antibody assays were manually read by the CLIA-CAP certified Oklahoma Medical Research Foundation Clinical Immunology Laboratory personnel using a Nikon Optiphot Fluorescence microscope with a HBO blub 100 w mercury lamp under the 20× objective.

### 2.3. Multiplex Bead-Based Autoantibody Assays

The Bio-Rad BioPlex 2200 (Bio-Rad, Hercules, CA) is a high-throughput, fully automated, serological analysis unit that utilizes multiplex bead technology for antibody detection. Dyed magnetic beads within the BioPlex 2200 ANA kit make possible the simultaneous detection of 13 different autoantibody specificities by using a method that has been previously described [26]. Ten of the detectable autoantibody specificities are commonly associated with SLE and target a variety of antigens including dsDNA, chromatin, ribosomal P, 60 kD Ro (SS-A 60), 52 kD Ro (SS-A 52), La (SS-B), Sm, Sm/RNP complex, nRNP A, and nRNP 68. Three other specificities were assessed (Scl-70, centromere B, and Jo-1) but were excluded from the majority of our analysis based on very low prevalence in this SLE cohort (2.3%, 3.7%, and 0.11%, resp.). Individual autoantibody responses are reported on a semiquantitative scale from 0 to 8, referred to as the Antibody Index (AI). This AI scale is set relative to calibrator, positive and negative control samples provided by the manufacturer. The defined positive cutoff value for each assay is then set to equal an AI of 1.0. However, anti-dsDNA results are reported in IU/mL and have a positive cut-off of 10.0 IU/mL per the manufacturer's recommendation. A sample is designated as ANA positive if detectable levels (AI ≥ 1.0 or IU ≥ 10.0) of antibody are found for any one of the analytes. 

### 2.4. Statistical Analysis

 These data represent a cross-sectional collection of SLE patient samples. Primary analyses used positive and negative classification of the autoantibodies and the SLE clinical criteria or subcriteria. Up to five members of a single family are included in these data with all hypothesis testing methods using an adjustment for these clustered family data (1803 patients and 1207 families). 

Descriptive statistics included percentages, frequencies, and means for demographic data and percentages and frequencies for autoantibodies and SLE clinical criteria or subcriteria. Heat map visualizations of the prevalence of each of the ACR criteria/subcriteria within each autoantibody positive population were generated using TIBCO Spotfire 4.0. Each distribution (positive or negative) of the collection of autoantibodies and the set of SLE clinical criteria and subcriteria were compared among the four ethnic groups using a generalized linear mixed model method incorporating multiple comparisons with adjustment of multiple testings (Bonferroni method). When ethnicity was found to be significant, multiple comparisons identified the pairwise statistically significant differences between the ethnic groups. Statistical significance was declared when an adjusted *P* value was less than alpha of 0.05. 

Conditional logistic regression modeling was used to examine associations between autoantibody specificities and SLE clinical criteria and subcriteria, with the ACR criteria and subcriteria serving as the outcome and the autoantibodies as the covariates. Univariate conditional logistic regression allowed for identification of associations between an autoantibody and a specific ACR SLE classification criterion. 

Multivariate conditional logistic regression modeling was performed to identify models demonstrating associations of covariates, autoantibodies, and sex with a specific outcome or grouped outcome. Interactions between the covariates as well as the potential association or confounding produced by sex were evaluated. Separate analyses were performed for each ethnic group. Odds ratios (ORs) and 95% confidence intervals (CI) were obtained as well as individual covariate statistical significance defined as a *P* value less than an alpha of 0.05. Confounding was considered present when at least a 20% difference in the OR estimate occurred when including sex in the model. An OR greater than 1.0 indicated a positive association of the antibody with the ACR criterion, where the odds of an outcome were higher for those patients with positive antibody results than for those with negative antibody results. Odds ratios of less than 1.0 indicated a negative association with the odds of the outcome lower for those patients with a positive antibody results compared to those with a negative autoantibody results. 

A Cochran-Mantel-Haenszel test (a chi-square test with stratification on family relationship) was used to examine SLE clinical manifestation prevalence in ANA positive compared to ANA negative individuals. All analyses were performed using SAS version 9.2 (SAS Institute Inc., Cary, NC) orSpotfire Decisionsite version 8.2.

## 3. Results

### 3.1. Cohort Demographics

This study examined a large, ethnically diverse, clinically heterogeneous cohort of 1,803 SLE patients (Table 1). Study participants met a minimum of 4 of 11 ACR clinical classification criteria [22, 23]. Our cohort consisted of 90% female SLE patients with an average age of 51.8 ± 15.4 and meeting an average of 5.15 ± 1.41 ACR classification criteria. EA SLE patients had the oldest average age at 43.2 ± 13.6 years; while HI SLE patients were the youngest at 37.9 ± 12.8 years. The age of the HI study participants was significantly lower than the EA and AA participants (*P* < 0.05). There was no statistical difference in the number of ACR classification criteria met or in the length of time from diagnosis to sample procurement between the self-reported ethnic groups. The only difference observed in medication usage was between AA and HI SLE patients. AA patients are more likely to be treated with biologic therapy (*P* = 0.0194); while HI patients were less likely to have no current treatment compared to both EA and AA patients (*P* = 0.0314).

### 3.2. Ethnic Differences in the Presentation of SLE ACR Criteria and Subcriteria

**Figure 1:**
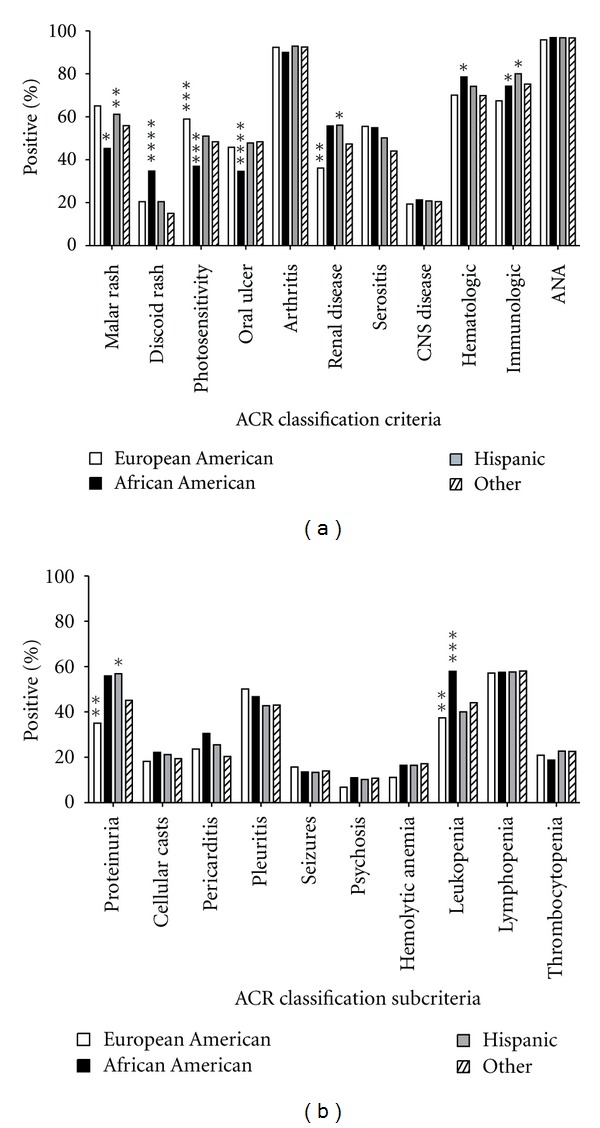
Prevalence of ACR classification criteria and subcriteria. ACR classification criteria (a) and subcriteria (b) prevalence by ethnicity are shown. European American (white bar), African American (black bar), Hispanic (dark grey bar), and other (multiracial/multiethnic, unknown, Asian, and American Indian, striped bar) are displayed. The most common classification criteria met are ANA, arthritis, hematologic, and immunologic. The most common ACR classification subcriteria is lymphopenia. Statistically significant differences (*P* < 0.05) are represented by stars. ****represents statistically different from all other racial groups; *represents statistical difference from EA; **represents statistical difference from AA; ***represents statistical difference from HI, and the striped star is difference from other.

The prevalence of specific ACR classification criteria was examined (Figure 1). The most prevalent criteria were ANA positivity, arthritis, hematological, and immunologic criteria. Ethnic differences in the prevalence of ACR criteria were observed (Figure 1(a)). Renal disorder and immunological disorders were significantly less prevalent in EA patients compared with other ethnic groups (*P* < 0.05). Although discoid rash and hematologic disorder were more prevalent in AA patients compared to both EA (*P* < 0.05) and HI (*P* < 0.05) patients, malar rash, photosensitivity, and oral ulcers were enriched in EA and HI (*P* < 0.05) patients. Immunological disorder was enriched in HI compared to EA (*P* < 0.05) SLE patients. To further dissect the disease profile difference among the different ethnicities (EA, AA, HI, and others) in this study, we also tested the prevalence differences of ACR classification subcriteria in all ethnicity groups (Figure 1(b)). As expected, enrichment of proteinuria was detected in AA and HI compared to EA (*P* < 0.05). The analysis also revealed that enrichment of hematologic disorder in AA compared to other two ethnicity groups was mainly due to the higher leukopenia prevalence in AA compared to EA and HI (*P* < 0.05). 

### 3.3. Initial Analysis Identified an Array of Autoantibody and Clinical Criteria Associations

**Figure 2:**
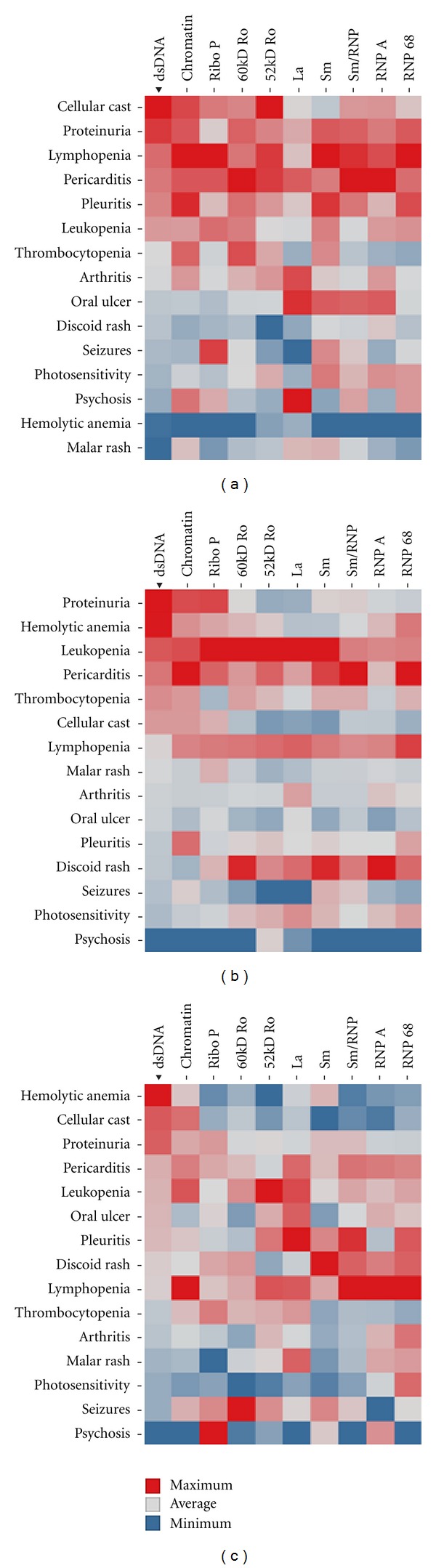
Association of SLE autoantibody prevalence and ACR criteria/subcriteria. Heat maps of the association between SLE autoantibody prevalence and ACR criteria/subcriteria for European Americans (a), African Americans (b), and Hispanic (c) are shown. Data is displayed as highest percent positive represented by the red square, average percent positive (gray square), and the minimum percent positive (blue square). The order of the rows is based on hierarchical clustering outcome of the clinical symptoms.

The initial Heat map of prevalence of each autoantibody in relation to each of the 15 ACR SLE clinical criteria is presented (Figure 2). The Heat map illustrated multiple autoantibody enrichments with renal disorder and hematologic disorder in EA, AA, and HI SLE patients. A select few autoantibodies were enriched in patients with mucocutaneous manifestation, within EA and HI patient populations. In particular, noticeable increased prevalence of La and Sm autoantibodies in patients with oral ulcers was observed. A significant enrichment of La and ribosomal P antibodies in patients with psychosis and seizure, respectively, was also observed. Compared to EA, AA and HI patients with discoid rash had increased positivity of Ro/La and Sm/RNP autoantibodies. 

Significant results from the initial univariate analyses are listed in Table 2. In EA, the most striking result was the association between hematologic criterion and five autoantibody specificities: anti-60 kD Ro, anti-52 kD Ro, anti-La, anti-Sm/nRNP, and anti-RNP 68 (Table 2). Anti-60 kD Ro, anti-52 kD Ro, and anti-RNP 68 were significantly enriched in patients with lymphopenia; while leukopenia was significantly associated with anti-52 kD Ro and Anti-ribosomal P antibodies. Anti-La responses were more common in SLE patients who did not have malar rash or proteinuria. Development of photosensitivity in EA patients is correlated with female sex (Table 2). 

In AA patients, anti-dsDNA, anti-chromatin, and anti-Sm/RNP are more commonly found in SLE patients with cellular casts; while lymphopenia was associated with antibodies against 52 kD Ro. Proteinuria showed significant association with sex with higher odds of development of proteinuria for females. Compared to EA, AA patients displayed a significant association between renal disorder-related subcriteria (proteinuria and cellular casts) and several autoantibodies; whereas EA demonstrated more individual antibody specificities correlated with lymphopenia than did AA. 

In the HI patients, the only significant associations between the presence of multiple antibodies and ACR criteria/subcriteria occurred in lymphopenia. In these patients, positive associations with lymphopenia were found with anti-RNP 68, anti-RNP A, and anti-Sm/RNP. Additionally, the presence of anti-chromatin antibodies was negatively associated with the development of hemolytic anemia. Univariate results in other ethnicity groups such as Asian and Native American were not significant at alpha = 0.05 level (not shown) likely due to small sample sizes.

Next we examined prevalence of SLE clinical criteria between ANA positive and ANA negative individuals as reported by the BioRad BioPlex 2200. No significant associations between ANA positive and SLE clinical criteria were observed in EA patients. However, AA ANA-positive patients were more likely to exhibit cellular casts (*P* = 0.0021), hematological disorder (*P* = 0.001), lymphopenia (*P* = 0.03), and immunological disorders (*P* = 0.0068). No difference was observed between the average number of antibodies in AA patients exhibiting and not exhibiting the specific SLE clinical criteria. The sample size for the HI patients was too small and, thus, the analysis could not be performed. Repeating this analysis utilizing ANA positivity as observed using indirect immunofluorescence found no significant differences in the prevalence of SLE clinical criteria in ANA-positive SLE patients of any ethnicity. Autoantibody frequency and SLE clinical criteria associations were further examined by multivariate analysis.

### 3.4. Leukopenia Is Associated with Anti-Ribosomal P Antibodies; while Lymphopenia Is Associated with Anti-52 kD Ro and Anti-RNP 68 Antibodies

Multivariate modeling results are shown in Table 3. Conditional logistic regression models were explored for all SLE clinical criteria with at least one significant univariate association. All 10 autoantibodies and sex served as covariates for these models. In the univariate analysis, both leukopenia and lymphopenia were correlated with a few autoantibodies in both EA and AA. However, in the multivariate model, leukopenia was associated with the presence of anti-ribosomal P in both EA and AA with both ORs greater than 3. This increased risk was almost 4-fold in EA. Anti-52 kD Ro and anti-RNP 68 antibodies were enriched in EA patients with lymphopenia; however, only anti-52 kD Ro autoantibodies were associated with lymphopenia in AA. Only one multivariate model with more than a single covariate was identified in HI patients. Anti-chromatin antibodies showed a positive association with lymphopenia; while anti-dsDNA antibodies had a negative association with this criterion. 

### 3.5. Individuals with Anti-Sm/RNP and Anti-dsDNA Antibodies Are Positively Associated with Cellular Casts in AA and Anti-Chromatin Antibodies are Positively Associated with Proteinuria in EA

Significant associations between renal disorder subcriteria (proteinuria and cellular cast) and an array of covariates including autoantibodies and sex were observed in both AA and EA. However, models revealed that covariates differed between AA and EA. In AA patients, development of cellular casts was associated with anti-dsDNA and anti-Sm/RNP antibodies. Sex was the only covariate correlated with proteinuria in the AA patients, in which females were more likely to develop proteinuria. Chromatin autoantibodies were enriched in EA SLE patients with proteinuria. Interestingly, while chromatin antibodies were positively associated with proteinuria, antibodies toward Sm and La were negatively associated. 

### 3.6. Sex and Anti-La Responses Are Associated with Mucocutaneous Criteria, while Anti-Sm/RNP and Anti-RNP Antibodies Are Enriched in European-American SLE Patients with Seizures

Associations between mucocutaneous clinical SLE presentation were assessed in EA and AA SLE patients. Anti-La antibodies were negatively associated with malar rash, while female sex was positively associated with higher odds of developing malar rash. Female sex in EA patients and anti-La antibodies in AA patients were correlated with photosensitivity. Interestingly, a negative association between anti-52 kD Ro antibodies and photosensitivity was observed in AA patients. An association between oral ulcers and anti-Sm/RNP antibodies was also only observed in AA (Table 3). Anti-Sm/RNP antibodies were enriched in EA patients with seizures (OR 5.33, 95% CI 1.15–24.8). No significant autoantibody and ACR criteria/subcriteria associations were observed in HI SLE patients. 

## 4. Discussion

 The goal of this study was to use multiplex autoantibody detection technology to determine associations between the presence of specific autoantibodies and classification criteria within a large, ethnically diverse cohort of SLE patients. We screened a panel of 10 autoantibody specificities that are often detected in SLE (dsDNA, chromatin, ribosomal P, 60 kD Ro, 52 kD Ro, La, Sm, Sm/RNP, RNP 68, and RNP A). Our study, as well as those of others, has described the BioPlex 2200 assay as a highly sensitive method for the detection of these autoantibody specificities [27–32]. Understanding associations between specific autoantibodies and SLE criteria as detected by this new methodology which is in widespread clinical use provides key insights into prognostic relevance for clinical application and may improve screening tests for diagnostic purposes.

Our study participants demonstrate a diverse, representative SLE patient population. While HI patients had a statistically lower age at participation (37.9 ± 12.8) than the other groups (EA 43.2 ± 13.6, AA 40.7 ± 12.4, and other 39.9 ± 12), this is consistent with the earlier age of SLE onset in the overall HI population [33, 34]. The most prevalent criteria in our cohort were ANA, arthritis, immunologic, and hematologic criteria, similar to those observed in previous studies [34–36]. 

Ethnic differences in autoantibody prevalence and association with ACR SLE classification criteria are observed in our study. We report a detailed association analysis between multiple autoantibodies and hematological disorder in our large Hispanic cohort, which has not been previously reported. We observed a significant difference between AA BioPlex 2200 ANA-positive and ANA-negative SLE patients. Hematological disorder (*P* = 0.001), lymphopenia (*P* = 0.030), and immunological disorder (*P* = 0.0068) were significantly enriched in ANA-positive AA patients. However, when a similar analysis was performed using indirect immunofluorescence, these associations disappeared. This is most likely due to the difference in specificity and sensitivity between the two assays. It is important to note that our EA patient group did have the lowest autoantibody prevalence for all tested specificities. Thus, the use of the BioPlex 2200 ANA may require the use of traditional autoantibody assays to confirm absence of ANA in this population subgroup. In AA patients, multiple autoantibodies associate with hematologic involvement in SLE. The associations between SLE autoantibody specificities and ACR criterion observed in our study confirm those observed in previous work [9, 33–35]. 

Our initial univariate conditional analysis demonstrates association between multiple autoantibodies. However, our multivariate adjusted conditional logistic regression analysis shows that antibodies to 60 kD Ro and RNP 68 are significantly independently enriched in EA patients with hematological disorders. In AA patients, anti-SM/RNP and anti-La antibodies are correlated with hematological disorder, while anti-RNP A antibodies alone are highly associated with hematological disorder in HI patients. Our results show that antibodies to ribonucleoproteins are highly prevalent in patients with hematological disorder. These results differ than those of Agmon-Levin et al. [2] and To and Petri [37]. Here, Sm/RNP antibodies were underrepresented in SLE patients with hematologic criteria. 

In our multivariate analysis, a significant association between anti-52 kD Ro, anti-ribosomal P, anti-RNP antibodies and the hematological ACR criterion is observed in EA and AA patients. Previous studies have identified correlations between anti-Ro and both lymphopenia and leukopenia or with lymphopenia alone and suggested a moderate association between anti-dsDNA and lymphopenia mostly in EA patient cohorts [12, 13]. A significant enrichment of anti-RNP 68 antibodies was observed in EA patients with lymphopenia. HI patients showed unique antibody associations with hematological ACR criterion. Interestingly, while antibodies to dsDNA were inversely associated with lymphopenia, anti-chromatin antibodies were directly associated with lymphopenia in HI patients. Our analysis has not only replicated the association between antibody to Ro and lymphopenia, but also revealed the inverse correlation between anti-dsDNA antibodies and lymphopenia in HI patients. However, differences in hematological criteria, especially luekopenia, may be due to benign ethnic neutropenia [38–41], a well-described characteristic that is not a manifestation of lupus, in some cases.

The association of dsDNA antibody specificity with renal disease has been widely demonstrated [8, 14, 42] and is also confirmed in our study. Interestingly, we have observed different distinct autoantibody associations with renal disease in EA and AA patients. The overall autoantibody associations with renal disease in EA are anti-chromatin and anti-Sm antibodies. Anti-dsDNA antibodies and female sex are more common in patients with renal disease; while anti-La antibodies are underrepresented in SLE patients with renal disease. These autoantibody association differences were maintained when examining renal disease subcriteria (proteinuria and cellular casts). In AA patients, female sex is associated with proteinuria; while anti-dsDNA and anti-Sm/RNP antibodies are correlated with cellular casts. The association between anti-Sm/RNP antibodies and renal criteria has not been previously described. In EA SLE patients, anti-Sm is mildly associated with proteinuria with odds ratio approaching 1.00, further studies are necessary to confirm this effect. No significant association between autoantibodies and renal involvement was observed in HI patients. Thus, our study suggests that anti-chromatin and absence of anti-La antibodies are the main predictors for renal involvement driven by prevalence of proteinuria in EA patients. However, the lack of autoantibody associations with cellular casts in EA SLE patients might be due to low numbers of EA patients being tested for cellular casts. Additionally, our study suggests that anti-dsDNA and anti-Sm/RNP antibodies are the strong correlates for renal disease as measured by cellular casts in AA patients. 

Ethnic differences in mucocutaneous manifestations of SLE are observed in our patient cohort. In our study population, EA patients show an increased prevalence of malar rash and photosensitivity; while AA patients exhibit increased frequency for discoid rash which is consistent with previous studies [43, 44]. Our autoantibody results indicate that in EA, malar rash is positively correlated with female sex; anti-La antibodies are negatively associated with malar rash in EA patients, while anti-La and anti-52 kD Ro antibodies are enriched in AA patients with photosensitivity. Several previously reported studies have observed an association between anti-RNP antibodies and photosensitivity [45, 46], which is not seen in our study. However, it is important to note that these two previous studies utilized a primarily Asian cohort [46, 47]. It is possible that this association also exists in our population, but the relatively small number of Asian study participants prevents this observation. 

The goal of this study was to examine associations between the prevalence of autoantibodies as detected by a Luminex bead-based assay (BioRad BioPlex 2200) and the occurrence of various clinical criteria in a large, ethnically diverse SLE patient cohort. The major findings of this paper identify associations between several antibody specificities and hematological disease, more specifically leukopenia and lymphopenia, and identify ethnic differences in autoantibody associations with renal subcriteria. Work is currently underway to better understand the mechanisms underlying these associations, particularly between leukopenia, lymphopenia, and autoantibody production. Autoantibodies play a crucial role in the diagnosis of SLE and a better understanding of the relationships between antibody prevalence and the presentation of other clinical criteria will further strengthen their prognostic implications.

## Figures and Tables

**Table 1:** Demographics of study participants.

Race/ethnicity	*N*(%)	Mean age (SD)	Mean number of ACR criteria (SD)	Female (%)
EA	836 (46.4%)	43.17 (13.63)	5.11 (1.4)	89
AA	618 (34.3%)	40.7 (12.4)	5.2 (1.4)	92
HI	255 (14.1%)	37.92 (12.82)	5.25 (1.55)	89
Other	93 (5.2%)	39.9 (12)	5.04 (1.4)	94

Total	1803 (100%)	51.76 (15.45)	5.15 (1.41)	90

EA: European American, AA: African American, HI: Hispanic, Other: mixed race/ethnicity, American Indian, Asian, and unknown.

**Table 2:** Univariate conditional logistic regression models within European-American, African-American, and Hispanic populations using ACR SLE criteria or subcriteria as the outcome and individual autoantibodies and sex as covariates.

Criteria/subcriteria	Covariates	European American		African American		Hispanic	
		Odds ratio (95% CI)	*P* value	Odds ratio (95% CI)	*P* value	Odds ratio (95% CI)	*P* value
	60 kD Ro	3.19 (1.55, 6.56)	0.0016				
	La	3.84 (1.43, 10.33)	0.0076				
Hematological disorder	52 kD Ro	2.99 (1.26, 7.07)	0.0128				
	Sm/RNP	2.28 (1.11, 4.70)	0.0257	2.64 (1.09, 6.37)	0.0315		
	RNP 68	3.92 (1.08, 14.27)	0.0379				
	RNP A					11.93 (1.53, 93.37)	0.0182

	52 kD Ro	2.10 (1.00, 4.38)	0.0489				
Leukopenia	Ribo P	3.77 (1.22, 11.60)	0.0207	3.16 (1.25, 8.00)	0.0154		
	Sm			2.31 (1.33, 6.31)	0.0072		

	60 kD Ro	2.26 (1.24, 4.15)	0.0082				
	52 kD Ro	2.78 (1.33, 5.79)	0.0064	2.90 (1.33, 6.31)	0.0072		
Lymphopenia	RNP 68	4.72 (1.30, 17.11)	0.0182			8.62 (1.05, 71.00)	0.0452
	RNP A					3.89 (1.07, 14.17)	0.0391
	Chromatin					3.13 (1.09, 8.97)	0.0334
	Sm/RNP					3.27 (1.09, 9.78)	0.0340

Hemolytic anemia	Chromatin					0.10 (0.01, 0.95)	0.0446

Renal disease	dsDNA			2.38 (1.15, 4.94)	0.0197		

Proteinuria	La	0.37 (0.15, 0.91)	0.0293				
	Sex			1.97 (1.04, 3.71)	0.0368		

	dsDNA			3.69 (1.46, 9.32)	0.0058		
Cellular casts	Chromatin			2.90 (1.27, 6.59)	0.0113		
	Sm/RNP			3.65 (1.55, 8.64)	0.0032		

Oral ulcer	Sm/RNP			1.97 (1.04, 3.71)	0.0368		

Photosensitivity	52 kD Ro			0.38 (0.16, 0.92)	0.0329		
	Sex	2.30 (1.00, 5.29)	0.0492				

Malar rash	La	0.43 (0.21, 0.89)	0.0234				
	Sex	3.17 (1.37, 7.33)	0.0069				

**Table 3:** Conditional multivariate adjusted logistic regression models within European-American, African-American, and Hispanic populations using ACR SLE criteria or subcriteria as outcome and individual autoantibodies and sex as covariates.

Criteria/subcriteria	Covariates	European American		African American		Hispanic	
		Odds ratio (95% CI)	*P* value	Odds ratio (95% CI)	*P* value	Odds ratio (95% CI)	*P* value
	60 kD Ro	3.31 (1.57, 6.99)	0.0017				
	RNP 68	4.37 (1.10, 3.74)	0.0361				
Hematological disorder	Sm/RNP			3.03 (1.21, 7.61)	0.0184		
	La			3.59 (1.07, 12.06)	0.0384		
	RNP A					11.93 (1.53, 93.37)	0.0182

Leukopenia	Ribo P	3.77 (1.22, 11.60)	0.0207	3.16 (1.25, 8.00)	0.0154		

	RNP 68	5.23 (1.40, 19.54)	0.0139				
Lymphopenia	52 kD Ro	2.95 (1.39, 6.29)	0.0050	2.90 (1.33, 6.31)	0.0072		
	dsDNA					0.18 (0.05, 0.69)	0.0124
	Chromatin					6.05 (1.70, 21.54)	0.0055

Hemolytic anemia	Chromatin					0.10 (0.01, 0.95)	0.0446

	Chromatin	2.56 (1.32, 4.95)	0.0054				
	La	0.33 (0.13, 0.88)	0.0273				
Renal disease	Sm	0.28 (0.10, 0.76)	0.0127				
	dsDNA			2.38 (1.11, 5.08)	0.0255		
	Sex			4.40 (1.17, 15.56)	0.0283		

	Chromatin	2.34 (1.23, 4.44)	0.0092				
Proteinuria	La	0.29 (0.11, 0.74)	0.0096				
	Sm	0.36 (0.14, 0.94)	0.0366				
	Sex			4.04 (1.11, 14.74)	0.0346		

Cellular cast	dsDNA			3.26 (1.25, 8.50)	0.0158		
	Sm/RNP			2.91 (1.24, 6.81)	0.0138		

Seizures	Sm/RNP	5.33 (1.15, 24.80)	0.0329				
	RNP A	0.013 (0.02, 0.67)	0.0156				

Oral ulcer	Sm/RNP			1.97 (1.04, 3.71)	0.0368		

Malar rash	La	0.42 (0.20, 0.89)	0.0233				
	Sex	3.23 (1.38, 7.55)	0.0068				

	52 kD Ro			0.07 (0.01, 0.53)	0.0103		
Photosensitivity	La			11.87 (1.37, 103.19)	0.0250		
	Sex	2.30 (1.00, 5.29)	0.0495				
